# Using a Resnet50 with a Kernel Attention Mechanism for Rice Disease Diagnosis

**DOI:** 10.3390/life13061277

**Published:** 2023-05-29

**Authors:** Mehdhar S. A. M. Al-Gaashani, Nagwan Abdel Samee, Rana Alnashwan, Mashael Khayyat, Mohammed Saleh Ali Muthanna

**Affiliations:** 1 College of Computer Science and Technology, Chongqing University of Posts and Telecommunications, Chongqing 400065, China; mr.mehdhar@gmail.com; 2 Department of Information Technology, College of Computer and Information Sciences, Princess Nourah bint Abdulrahman University, P.O. Box 84428, Riyadh 11671, Saudi Arabia; nmabdelsamee@pnu.edu.sa; 3 Department of Information Systems and Technology, College of Computer Science and Engineering, University of Jeddah, Jeddah 23218, Saudi Arabia; mkhayyat@uj.edu.sa; 4 Institute of Computer Technologies and Information Security, Southern Federal University, 347922 Taganrog, Russia; muthanna@sfedu.ru

**Keywords:** rice disease classification, self-attention mechanism, agriculture imaging

## Abstract

The domestication of animals and the cultivation of crops have been essential to human development throughout history, with the agricultural sector playing a pivotal role. Insufficient nutrition often leads to plant diseases, such as those affecting rice crops, resulting in yield losses of 20–40% of total production. These losses carry significant global economic consequences. Timely disease diagnosis is critical for implementing effective treatments and mitigating financial losses. However, despite technological advancements, rice disease diagnosis primarily depends on manual methods. In this study, we present a novel self-attention network (SANET) based on the ResNet50 architecture, incorporating a kernel attention mechanism for accurate AI-assisted rice disease classification. We employ attention modules to extract contextual dependencies within images, focusing on essential features for disease identification. Using a publicly available rice disease dataset comprising four classes (three disease types and healthy leaves), we conducted cross-validated classification experiments to evaluate our proposed model. The results reveal that the attention-based mechanism effectively guides the convolutional neural network (CNN) in learning valuable features, resulting in accurate image classification and reduced performance variation compared to state-of-the-art methods. Our SANET model achieved a test set accuracy of 98.71%, surpassing that of current leading models. These findings highlight the potential for widespread AI adoption in agricultural disease diagnosis and management, ultimately enhancing efficiency and effectiveness within the sector.

## 1. Introduction

The cultivation of rice, a fundamental food crop, is confronted with a multitude of obstacles presented by various diseases, which have a considerable impact on its development and productivity. According to the Food and Agriculture Organization of the United Nations (FAO), the annual global crop yield losses resulting from diseases and pests are estimated to amount to approximately USD 220 billion [1]. The prompt detection and precise diagnosis of plant diseases are of utmost importance in order to mitigate associated financial damages. Farmers frequently overlook initial indications of disease or postpone seeking medical attention owing to misinterpretation, primarily as a result of insufficient specialized expertise and resources.

Increased accessibility to smartphones and advanced digital cameras has facilitated the process of capturing visual representations of afflicted agricultural crops. Simultaneously, progressions in computer vision technologies have facilitated the processing of images and the diagnosis of diseases in diverse research endeavors [2,3,4]. Pesticides are commonly employed as the principal approach for the prevention and management of diseases in the field of agriculture. Precise diagnostic results are essential in directing the utilization of pesticides, as their overuse is a significant contributor to the deterioration of the environment [5]. Therefore, it is imperative to accurately and promptly diagnose diseases.

The conventional method of diagnosing rice leaf diseases is known to be arduous and time-intensive. Consequently, there has been an increasing inclination towards utilizing computer-assisted diagnostics as a pivotal instrument for the detection and classification of rice ailments. The exceptional generalization capabilities of convolutional neural networks (CNNs), which belong to the category of deep neural networks (DNNs), have been demonstrated in various image-processing studies. For instance, a deep residual network based on attention mechanisms was proposed to identify viruses in tomato leaves [6]. Several classifiers based on deep learning have demonstrated their efficacy in identifying images of rice diseases [7,8]. Attention modules have been found to improve a model’s effectiveness in capturing relevant dependencies [9,10,11]. The selection of suitable loss and activation functions is a critical factor in attaining favorable outcomes with deep networks, as indicated by sources [12,13]. The utilization of deep neural network (DNN) methodologies in the field of rice disease diagnosis has not been extensively explored, resulting in a dearth of models that are tailored to the classification of rice diseases, despite the swift advancements in this area.

The objective of this investigation was to perform a comprehensive examination of the disease patterns present in rice leaves by utilizing a deep learning algorithm. Our proposal involves the implementation of a linear-kernel attention-based mechanism that aims to enhance the capacity of deep neural networks to selectively attend to crucial features. In contrast to prior research, our model takes into account distant connections within feature maps, which constitutes a notable progression in the domain. In order to effectively train deep learning models, a significant quantity of rice leaf images is typically necessary to extract diagnostically relevant features. The kernel attention method that we propose aims to improve the performance of the deep neural network model’s process of learning by directing the model’s attention toward the extraction of features that contain more relevant information. The kernel attention mechanism is a relatively new concept that was recently presented in the work that was conducted by Li et al. [14] for the segmentation of remote-sensing images. Imagery obtained by remote sensing can be used to keep track of and locate new urban areas as they emerge as a result of ongoing urbanization. The work presented here applies the same principle of extracting the most useful feature, utilizing kernel attention for a different application, namely, the classification of rice diseases rather than their segmentation. Comprehending the learning mechanisms of neural networks and effectively visualizing this process are of utmost importance. The utilization of feature visualization aids in illuminating the viewpoint of the model regarding the visual surroundings. It also provides insights into the manner in which a pre-existing convolutional network extracts features as well as desirable characteristics, such as feature composition and class discrimination, which become more pronounced as the network layers increase. The utilization of visualization techniques has been demonstrated to facilitate the process of model debugging, ultimately resulting in improved outcomes [15]. The contributions of the present study are as follows:

- A neural network is developed that utilizes self-attention based on kernel attention linear complexity (SANET) for the purpose of classifying various types of rice diseases.
- The SANET model is designed to hierarchically aggregate contextual data using multiscale kernel attention, thereby enabling the inference of global contextual dependencies.
- A novel self-attention mechanism is proposed that incorporates kernel attention to reduce high computational demand with linear complexity.

This paper is organized as follows: Section 2 presents an overview of deep learning models and their application in rice disease studies. Section 3 provides an in-depth review of the proposed model and the impact of kernel attention in feature extraction and long-range dependencies. Section 4 discusses the experimental results and evaluates the deep learning model’s performance. Finally, Section 5 concludes the research findings and outlines avenues for future work.

## 2. Related Work

In recent years, many unique deep-learning algorithms have been introduced and put into use for the purpose of identifying rice diseases. A new method for detecting diseases in rice was proposed by Yang Lu et al. [16] using deep CNN techniques. The researchers used a dataset with 500 images of both healthy and diseased rice leaves and stems. These images were captured in an experimental rice field that was infected with 10 different types of rice infection. When put through a 10-fold cross-validation method, the accuracy of the proposed CNNs-based model was confirmed to be 95.48%.

State-of-the-art architectures, such as Inceptionv3 and VGG16, were adapted by Rahman et al. [17] for the classification and detection of rice diseases. The results from their experiments proved the models’ value when used with real-world data. The proposed two-stage small CNN architecture was compared to memory-efficient solutions such as MobileNet [18], NasNet Mobile [19], and SqueezeNet [20]. As large-scale architectures are incompatible with mobile devices, this change was made. After making significant reductions in the size of the model, they were still able to achieve the desired level of accuracy (93.3%).

With the assistance of several experts, Liang et al. [21] have published a dataset for the classification of rice leaf diseases. In addition to this, they suggested using a CNN as the basis for an approach to feature extraction and disease classification. The results of their experiments demonstrated that the high-level features derived from convolutional neural networks possessed superior discriminative capabilities compared to those derived from Haar-WT (wavelet transform) and local binary pattern histograms (LBPH). According to the results of their research, the hybrid CNN and SVM that they proposed, which they called support vector machines, had greater accuracy and a larger value of the AUC, area under the curve, than conventional methods such as Haar-WT + SVM and LBP + SVM.

Ramesh and Vydeki made use of several image-processing techniques in order to reduce the amount of reliance placed on farmers to ensure the safety of agricultural products [22]. They proposed an algorithm for the classification of paddy leaf diseases utilizing an improved deep neural network in conjunction with the Jaya algorithm. Their photographs of rice plant leaves included healthy plants as well as those with bacterial brown spots, blights, blast diseases, and sheath rot. These images were taken directly from the agricultural field. During the preprocessing phase, the RGB images were converted to HSV images to eliminate the background, and then, based on the hue and saturation components of the images, binary images were extracted to distinguish sick tissue from healthy tissue. In order to categorize the infected leaves, they developed a deep neural network using the Jaya optimization algorithm (DNN-JOA). They were able to classify blast-afflicted leaves, bacterial blight leaves, sheath rot leaves, brown spot leaves, and healthy leaf images with accuracies of 98.9%, 95.78%, 92%, 94%, and 90.57%, respectively. RiceTalk [23] is a project that was developed by Chen et al. that makes use of non-image Internet of Things sensors to detect rice blasts. RiceTalk was based on an Internet of Things platform for soil agriculture and was able to achieve an accuracy of 89.4% on rice blast disease.

Until the advent of deep learning, machine vision systems were often implemented using statistical machine learning techniques, notably, those based on the fields of supervised and unsupervised learning. Naive Bayes (NB), discriminant analysis (DA), support vector machines (SVMs), and k-nearest neighbors (kNN) are some of the modern methods studied by Rehman et al. [24]. The study provided an in-depth examination of how these methods are currently being used in a range of agricultural domains, drawing the conclusion that different methods should be used for different purposes while acknowledging their limitations. On top of that, Duong-Trung et al. [25] used transfer learning to categorize rice discoloration disease, which was previously thought to be a major threat to rice production. To improve the efficiency of deep models that are already familiar with low-level characteristics, transfer learning employs weights that are pre-trained on data from different domains. The results of this study used an In-ceptionV3 model pre-trained on ImageNet to achieve an accuracy of 88.18% in classification, similar to how Shrivastava et al. [26] used transfer learning for a convolutional neural network to categorize rice plant diseases (CNN). Using a training/testing split of 80/20, the suggested model achieved a classification accuracy of 91.368%.

Chung et al. [27] developed a non-destructive method using machine vision to distinguish between infected and healthy seedlings after three weeks of growth. Their work centered on identifying bakanae disease, a seed-borne threat to rice. Infected plants either fail to thrive or produce fruitless panicles. In order to quantify the morphological and color characteristics of the infected and control seedlings, pictures were collected with the aid of flatbed scanners. Support vector machine (SVM)-based classifiers were utilized. Additionally, it was suggested that a genetic algorithm may be employed to find the optimal combination of required and optional model parameters. Their method had an accuracy of 87.89% in distinguishing between healthy and diseased seedlings. To properly evaluate the efficacy of a deep learning model, a thorough analysis of the features it employs is required. This was the motivation behind the study’s implementation of a method for visualizing the feature maps of rice disease images [16]. In a subsequent study [17], Rahman and colleagues expanded on this method by extracting and retaining information from the deep learning model’s early and intermediate layers to classify various forms of rice disease.

Tai et al. [28] used two ViT models in tandem to handle images of varying resolutions, and techniques [29,30] have recently implemented the ViT (ViT-B16 with 16 attention blocks and ViT-B32 with 32 attention blocks) without altering the images in any way. Some of these studies examined the specific disease that was affecting a plant [28,30], whereas others focused on the categorization of plants rather than the diseases that were affecting them. However, as additional plant species and disease strains emerge, it becomes increasingly challenging to address the issue. However, using such a deep neural network for the categorization of plant diseases may be excessive, and simpler models may be able to perform adequately well in some situations. Despite the benefits of the ViT’s performance, it is highly impractical to use transformer-based models for leaf classification. Thus, in this paper, we propose combining the self-attention power of transformers with Resnet50 [31]. We used linearly complex kernel attention to capture long-term dependencies across many resolutions, which directly reduced the model’s memory footprint.

The study conducted by Wang et al. [32] was another effort that is extremely comparable to ours and also very important to our approach. They devised the attention-based depth-wise separable neural network with Bayesian optimization, which is abbreviated as ADSNN-BO, in order to detect rice diseases in a timely and accurate manner. The foundation of their model is a MobileNet pre-trained CNN that incorporates an attention mechanism. In addition, the Bayesian optimization method is utilized in order to fine-tune the hyper-parameters. It has been determined that their model is 94.65% accurate. Additionally, their technique helps improve interpretability by offering feature analysis through the utilization of an activation map and filters visualization. In order to highlight the contrasts between our research and Wang et al.’s model, we would like to emphasize that although Wang et al. employed a MobileNet pre-trained CNN model in the classification of input images, we have classified our images using Resnet50 in our system. In addition, our model is intended to hierarchically aggregate contextual input through the use of multiscale kernel attention; yet, Wang et al.’s model only added an attention-augmented layer to the MobileNet pre-trained model. In addition to this, we are including a novel self-attention mechanism in our model, which also makes use of kernel attention, with the goal of lowering the high computing demand while maintaining linear complexity.

## 3. Proposed Method

### 3.1. Dot Product Attention

**Figure 1:**
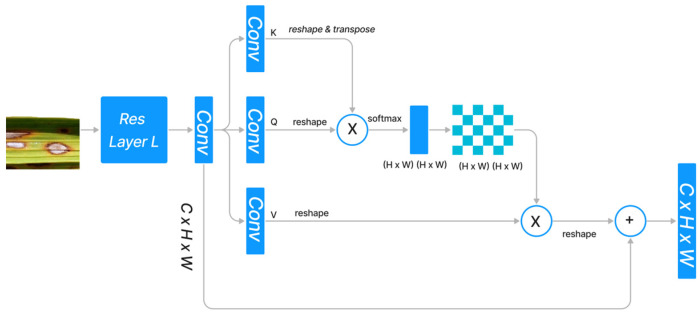
Standard Dot Product Attention Architecture.

The standard dot product attention architecture is depicted in Figure 1. Given the features $\left[x_{1},x_{2},x_{3}\ldots .,\;x_{n}\right]\in \;{\mathbb{R}}^{N\;\times \;C}$, dot-product attention generates three projected matrices, i.e., query matrix *Q*, key matrix *K*, and value matrix *V*, using $W_{q}\in \;{\mathbb{R}}^{N\;\times \;C}$*,* where N  indicates the size of the input and C denotes the input channels.

(1) $$Q=XW_{q}\in \;{\mathbb{R}}^{N\;\times \;D_{k}}$$

(2) $$K=XW_{k}\in \;{\mathbb{R}}^{N\;\times \;D_{k}}$$

(3) $$V=XW_{v}\in \;{\mathbb{R}}^{N\;\times \;D_{k}}$$

$D\left(.\right)$ Indicates the dimensions of dot product. We represent both *Q* and *K* with the same symbol because they are supposed to have the same shape.

In order to calculate the similarity between the *i*th query feature vector $\left(q_{i}^{T}\right)\;\epsilon \;{\mathbb{R}}^{D_{k}}$ and the *j*th key feature vector $k_{j}\;\epsilon \;{\mathbb{R}}^{D_{k}}$, we used the normalization function $p\left(q_{i}^{T}k_{j}\right)\;\epsilon \;{\mathbb{R}}^{1}$. Since the query feature and the key feature are frequently generated by distinct layers, the similarities among $p\;\left(q_{i}^{T}\right)$ and ($p\;\left(q_{j}^{T}\right)\;$) are typically asymmetrical. The dot product attention module determines the value at the position i  by performing a weighted sum across all positions, where each position’s value feature is assigned a weight based on its similarity to all other positions.

(4) $$D\left(Q,K,V\right)=p\left(QK^{T}\right)V$$

(5) $$p\left(QK^{T}\right)=softmax\left(QK^{T}\right)$$

softmax indicates that the softmax operation is carried out along each column of the matrix $QK^{T}$. $p(QK^{T}$) denotes the similarity between all pairs of locations. The memory complexity and computational complexity are both $O\left(N^{2}\right)$ because $Q\;\in \;{\mathbb{R}}^{D_{k}\times \;N}$ and $K^{T}\in {\mathbb{R}}^{D_{k}\times \;N}$, respectively, and because the product $Q\in {\mathbb{R}}^{N\;\times \;N}$ $K^{T}$ $\in {\mathbb{R}}^{N\;\times \;N}$. Therefore, the dot-high product’s resource requirement severely restricts its applicability to high-dimension inputs. Altering the softmax is a strategy, while reframing the attention via the lens of the kernel is another approach. Figure 1 depicts the design of the dot-product attention mechanism, which integrates the refined features with the original input through a skip link after capturing the long-range context information from feature maps produced by CNN.

### 3.2. Dot Product Based on Kernel Attention

As demonstrated in Equation (2), the *i*th row of the result matrix obtained by dot-product attention may be written as follows:

(6) $$D{\left(Q,K,V\right)}_{i}=\frac{\sum_{j=1}^{N}e^{q_{i}qk_{j}}v_{j}}{\sum_{j=1}^{N}e^{q_{i}qk_{j}}}$$

where *i* is an iteration identifier and softmax is the softmax normalization function.

From Equation (4), we can deduce that the dot-product attention mechanism works by averaging the weights assigned to the value matrix *V* using $e^{q_{i}qk_{j}}$, using the similarity measure $sim\left(q_{i},k_{j}\right)$ = $e^{q_{i}qk_{j}}$ between query matrix *Q* and key matrix *K*. Therefore, we can generalize Equation (4) by substituting a generic form for the softmax function, as follows:

(7) $$D{\left(Q,K,V\right)}_{i}=\frac{\sum_{j=1}^{N}sim\left(q_{i},k_{j}\right)v_{j}}{\sum_{j=1}^{N}sim\left(q_{i},k_{j}\right)},\;sim\left(q_{i},k_{j}\right)\geq 0$$

Specifically, $sim\left(q_{i},k_{j}\right)$ is the function that assesses the degree of similarity between $q_{i}$ and $k_{j}$. Under the assumption that $sim\left(q_{i},k_{j}\right)$ = $e^{q_{i}qk_{j}}$, we have Equation (5), which is equivalent to Equation (4). Simultaneously, we can write $sim\left(q_{i},k_{j}\right)$ =$\varphi {\left(q_{j}\right)}^{T}\;\emptyset \left(k_{j}\right)$, where $\varphi \left(.\right)$ and $\emptyset \left(.\right)$ are kernel smoothers [33]. Therefore, the inner product space can be described as $\langle \varphi \left(q_{j}\right)\;\emptyset \left(k_{j}\right)\rangle$.

Equation (4) can then be further rewritten as

(8) $$D{\left(Q,K,V\right)}_{i}=\frac{\sum_{j=1}^{N}\varphi {\left(q_{i}\right)}^{T}\emptyset \left(k_{j}\right)v_{j}}{\sum_{j=1}^{N}\varphi {\left(q_{i}\right)}^{T}\emptyset \left(k_{j}\right)}$$

which can be further simplified as

(9) $$D{\left(Q,K,V\right)}_{i}=\frac{\varphi {\left(q_{i}\right)}^{T}\sum_{j=1}^{N}\emptyset \left(k_{j}\right)v_{j}}{\varphi {\left(q_{i}\right)}^{T}\sum_{j=1}^{N}\emptyset \left(k_{j}\right)}$$

$K\in {\mathbb{R}}^{D_{k}\;\times \;N}$ and $V^{T}\;\in {\mathbb{R}}^{D_{k}\;\times \;N}$, which significantly lowers the level of complexity with the dot-product attention method.

### 3.3. Kernel Attention Mechanism

We took $\varphi \left(q_{j}\right)=\emptyset \left(k_{j}\right)=swish\left(.\right)$ where

(10) $$swish\left(.\right)=x*sigmoid\;\left(x\right)$$

The reason we chose Swish(.) over ReLU(.) is because when the input is somewhat negative, then the nonzero property of Swish can allow the attention mechanism to avoid zero gradients. The function can be implemented as

(11) $$sim\left(q_{i},k_{j}\right)=swish\;{\left(q_{i}\right)}^{T}\;swish\;\left(k_{j}\right)$$

Consequently, Equation (5) may be written as

(12) $$D{\left(Q,K,V\right)}_{i}=\frac{swish\;{\left(q_{i}\right)}^{T}\;\sum_{j=1}^{N}swish\;\left(k_{j}\right)v_{j}^{T}}{swish\;{\left(q_{i}\right)}^{T}\sum_{j=1}^{N}swish\;\left(k_{j}\right)}$$

which can be further simplified as

(13) $$D\left(Q,K,V\right)=\frac{swish\;\left(Q\right)\;swish\;(K^{T})\;V}{swish\;\left(Q\right)\sum_{j}swishK_{i,j}^{T}}$$

Given $\overset{N}{\underset{j=1}{\sum }}swish\;\left(k_{j}\right)v_{j}^{T}$ and $\overset{N}{\underset{j=1}{\sum }}swish\;\left(k_{j}\right),\;$ the time and memory complexity of the proposed kernel attention technique based on (11) is merely O(N), as each query can be calculated and reused.

### 3.4. Attention Network

**Figure 2:**
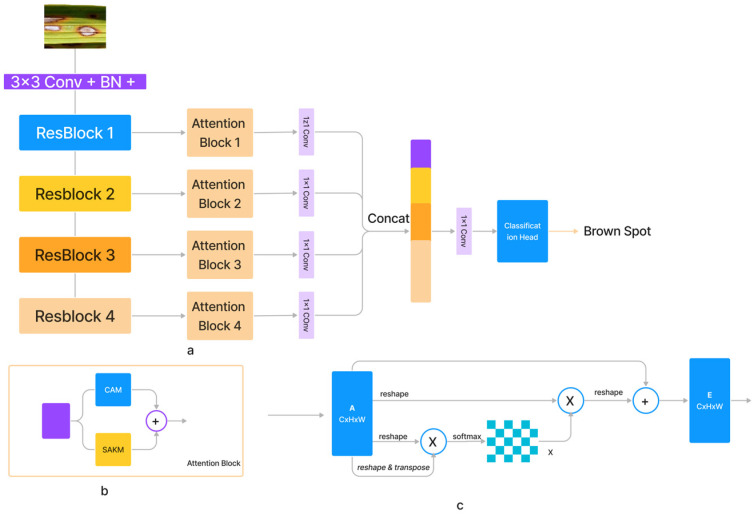
The architecture of (**a**) proposed SANET, (**b**) SAKM, (**c**) CAM Block.

With increasing input size N=H×W, the computational complexity of the dot-product attention mechanism increases exponentially. We provided a self-attention module for the spatial dimension based on kernel attention to address this problem (SAKM). In the vast majority of instances, the number of input channels *C* was significantly lower than the number of pixels *N* in the feature maps along the channel dimension. Therefore, channel softmax function complexity was reasonable at $O\left(C^{2}\right)$ (3). The channel attention mechanism (CAM) [34] based on the dot-product was thus implemented (as shown in Figure 2). Comparable residual relationships existed between the SAKM, CAM, and dot-product attention mechanism, which was the direct sum of output and input features. SAKM and CAM were applied to produce an attention block that improved the discriminative capabilities of the generated feature maps for each layer. Both the SAKM and the CAM utilized the ResBlock’s created characteristics to hone in on the data’s location and channel, respectively. Figure 2 shows that the output of the attention block was generated by concatenating the revised feature maps. Figure 2 presents a representation of the proposed SANET architecture (a). These feature maps were generated using an ImageNet-pre-trained ResNet-50. The feature maps generated by Resnet50’s convolutional layers were further augmented using attention blocks. The characteristics from the attention blocks were combined, and the resulting output was then processed through a classification head to forecast the class.

## 4. Dataset and Experimental Settings

### 4.1. Dataset Description

**Figure 3:**
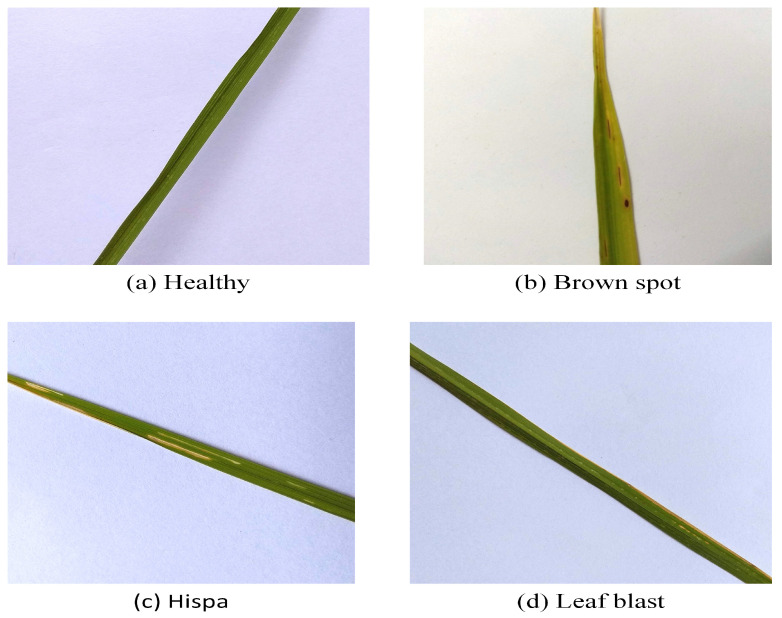
Some samples from the rice disease dataset. Healthy (**a**) and diseased leaf samples from the dataset. Three disease types in this study: (**b**) brown spot, (**c**) rice hispa damage, and (**d**) leaf blast.

For this study, we focused on the three most common types of rice disease: brown spot, rice hispa damage, and rice leaf blast. The foundation of the common manual diagnostic process is the visual representation of symptoms. Brown spots, also known as age spots, are flat, dark brown lesions that are typically spherical or oval in shape and surrounded by a yellow halo. A lesion’s round shape is maintained regardless of its size, and it always has a gray, necrotic center and a reddish-brown to dark-brown border. Rice hispa destroys the epidermis on the upper surface of the leaf blades. The disease eats away at the leaf tissue. Plants lose health and vitality when subjected to extreme stress. The damage caused by hispa can be easily determined if the bug is spotted on a rice leaf. Rice leaf blast can cause lesions ranging from tiny black dots to larger oval patches with a reddish rim and a gray or white center. Spots grow longer and more diamond or baroque in shape, with sharply pointed ends and gray, lifeless centers surrounded by thinner rings of reddish brown. There were some completely healthy instances of rice among the 2370 leaf samples we analyzed [35]. Table 1 illustrates the number of samples for each disease category. From each disease category, 100 samples were selected for training and testing. In Figure 3, Some samples from the rice dataset are shown.

### 4.2. Experimental Setting

As the backbone, we chose ResNet-50, which had already been trained on ImageNet. The Adam optimizer was configured with a learning rate of 0.0001 with 32 batch sizes. All experiments were carried out on a single NVIDIA 3090 GPU with 24 gigabytes of VRAM. For quantitative evaluation, cross-entropy loss combined with backpropagation was used to measure the gap between predicted classes and labels.

(14) $$\left(p,y\right)=-y\log\left(p\right)-\left(1-y\right)\log\left(1-p\right)$$

*p* represents the prediction and *y* indicates the label.

### 4.3. Evaluation Metrics

The performance of SANET on the rice disease classification dataset was measured using the classification accuracy (14) and F1 score (15), which were computed on the cumulative confusion matrix. An F1 score is used to evaluate models in machine learning by measuring how well they perform. Essentially, it averages a model’s precision and recall.

(15) $$Classification\;Accuracy=\frac{\sum_{k=1}^{N}TP}{\sum_{k=1}^{N}TP+FP+TN+FN}$$

(16) $$F1=2\times \frac{precision\;\times \;recall}{precision+recall}$$

$TP_{k},FP_{k},TN_{k},\;and\;FN_{k}$ represent the true positives, false positives, true negatives, and false negatives, respectively. Classification accuracy was calculated for all classes.

### 4.4. Experimental Results and Analysis

We conducted experiments using a dataset of 400 images labeled with four disease categories. The proposed model was compared against a number of other deep learning models, including VGG16 [36], ResNet50 [31], Dense-Net121 [37], MobileNetV1 [18], InceptionV3 [38], Xception [39], and ViT [33], as shown in Table 2. Each CNN model with pre-trained weights was tested on ImageNet using five-fold cross-validation and the same architecture as the original. Based on the performance metrics discussed in the evaluation criteria, Table 3 displays the assessed results. Each measurement has both its mean and standard deviation listed in the table. The five-fold cross-validation results underpinned these values. Characters in bold represent the deep learning models with the highest ratings from the evaluations (see Table 4 for details).

**Figure 4:**
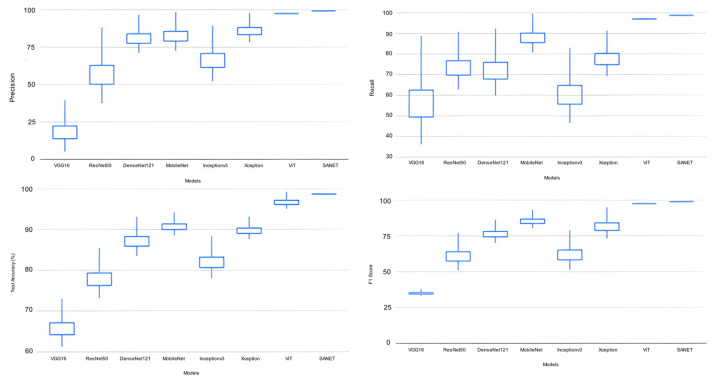
Classification Performance Variation.

**Figure 5:**
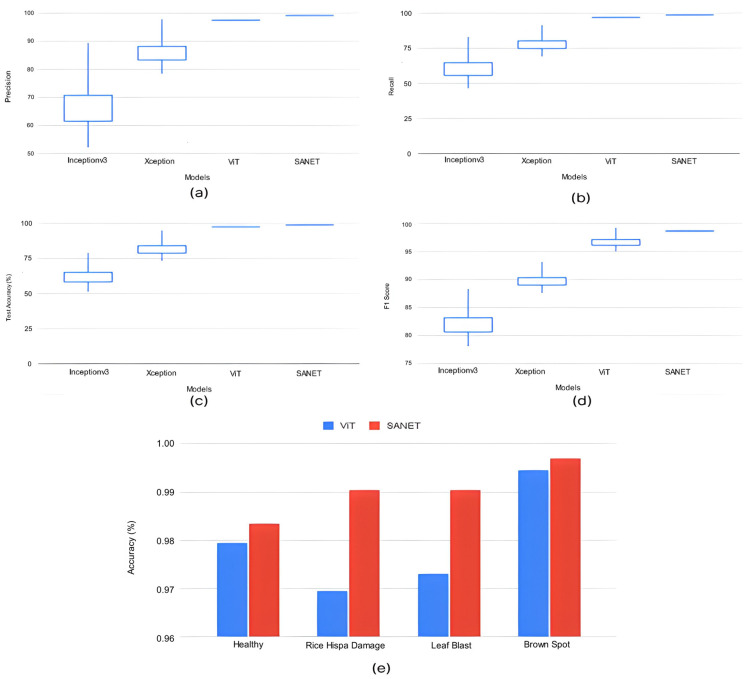
(**a**–**d**) Classification Performance Variation in Top Deep Learning Models. (**e**) Accuracy variation in top performance models for all classes.

The proposed SANET model had the lowest variability in its performance while still achieving the highest test accuracy, recall, precision, and F-1 score. As shown in Table 4, the SANET improved upon the ViT by 1.6% in precision, 1.2% in F-1 score, and 1.55% in accuracy while using the same 28.62 million parameters. We also evaluated the relative merits of the three most accurate models (ViT, MobileNet, and Xception) in comparison to SANET’s overall performance. Figure 4 and Figure 5 show box plots representing the model’s accuracy and F-1 score. To classify images consistently with a lower error rate, the SANET outperformed other state-of-the-art models across the board for each type of rice disease. According to our analysis of multiple classes of rice diseases, the leaf blast disease is the most difficult to categorize. The leaf blast disease is the most difficult to classify when compared to other types of diseases discussed in this study, such as brown spot and rice hispa damage. This is because the images in each class were organized in a specific way. Brown spots, as described in Section 4, are flat, dark brown lesions that are often spherical or oval in shape and encircled by a yellow halo. The occurrence of brown spots is not constrained to specific regions; they have the propensity to manifest on any part of the plant’s anatomy. Rice hispa is responsible for the death of the epidermis that is found on the upper surface of leaf blades. The leaf tissue is consumed by the illness as it spreads. When under severe stress, plants experience a decline in their overall health and vitality. If a hispa is found on a rice leaf, the extent of the damage that it has inflicted can be ascertained with relative ease. Rice leaf blasts, on the other hand, can create lesions that range from very small black spots to larger oval patches with a ruddy ring and a gray or white center.

Two methods are used to assess the quality of the characteristics extracted by the proposed model.

(1) Activation maps
(2) Filter visualization.

**Figure 6:**
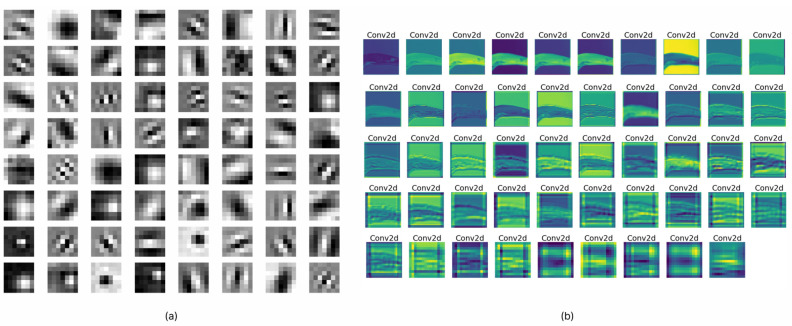
(**a**) Filter visualizations. (**b**) Feature maps activations.

An activation map is widely regarded as a technique for visualizing data and producing a diagram that shows the activation levels. To generate the activation maps, we needed to load the weights of the best-performing network. The test sample of rice images was passed as an input to the network. Following the execution of each layer, the output indicates the kind of input that activates the layer to its fullest extent. A number of carefully chosen activation maps were constructed, as shown in Figure 6a. The figure illustrates how the model treated the various diseases that might affect rice. When attempting to diagnose brown spot disease, for instance, the network was able to successfully capture and extract the spot pattern. In addition, activation maps for each of the filters that were contained inside a single layer are shown in Figure 6b. According to the observations, it is evident that the majority of filters were activated in relation to the pattern of each disease, and it is also evident that different types of patterns were activated for different disease categories. If the generated feature maps were blank, this indicated that the network was not able to localize the disease in the given test image. These patterns were almost certainly made up of intricate shapes that were not in the original image.

**Figure 7:**
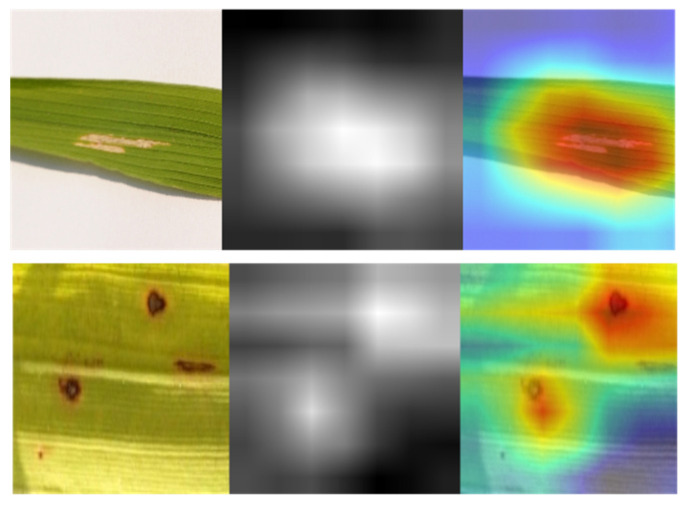
Class Activation Maps from SANET.

Filter visualization of a deep learning model differs from activation maps in that it may depict filters, which are the network’s weights obtained from training. Figure 7 illustrates the filters of SANET’s several convolutional layers that treat rice diseases. As shown in the figure, the uppermost levels of a model’s neural network often provide evidence of its ability for learning. Since the bottom half of the model tended to amass unnecessary visual patterns and information, top-layer filters were frequently intelligible and capable of evaluating a model’s performance. In addition, one may claim that SANET is sensitive to the pattern of brown spot spots. The filters were more identifiable as a result of the attention process. In addition, the attention process enabled SANET to locate the illness in the provided picture, as illustrated in Figure 7. This localization was entirely unsupervised and required no annotations.

## 5. Conclusions

An approach to the classification of rice leaf diseases that is simple and straightforward is proposed in this article. We devised a method of aggregating contextual features at various levels of the encoder by integrating kernel attention within self-attention modules that had linear complexity. This allowed us to extract semantic information from several layers. In a variety of experiments, the performance of our proposed SANET model was superior to that of previously developed deep learning models. In addition, a feature assessment, activation maps, and filter operations were carried out in order to highlight the performance of the model. On the rice disease dataset, our proposed model outperformed other state-of-the-art evaluated models by a significant margin, with only a small amount of variation in classification performance.

## Figures and Tables

**Table 1:** Rice disease dataset information by classes.

Category	Number of Samples
Rice Hispa Damage	565
Leaf Blast	779
Brown Spot	523
Healthy	501
Total	2368

**Table 2:** Comparison with other deep learning algorithms.

Model	Precision ± SD	Recall ± SD	F-1 Score ± SD	Test Accuracy ± SD
Xception	88.2 ± 9.68	80.4 ± 11.06	84.2 ± 10.76	90.38 ± 2.74
ResNet50	62.8 ± 25.24	76.8 ± 14.04	64.0 ± 12.98	79.32 ± 6.12
MobileNet	85.6 ± 12.95	90.2 ± 9.28	86.8 ± 6.22	91.36 ± 2.82
VGG16	22.3 ± 17.08	62.5 ± 26.30	35.5 ± 2.38	67.12 ± 5.84
Inception V3	70.8 ± 18.58	64.8 ± 18.19	65.2 ± 13.77	83.20 ± 5.08
DenseNet121	84.0 ± 12.67	76.0 ± 16.31	78.2 ± 7.98	88.27 ± 4.77
ViT	97.6 ± 0.007	97.1 ± 0.26	97.6 ± 0.12	97.15 ± 2.07
SANET	**99.2 ± 0.006**	**98.8 ± 0.002**	**98.9± 0.06**	**98.7± 0.16**

**Table 3:** Classification performance comparison with deep learning algorithms based on three categories.

Model			Classification Category	
	Brown Spot	Healthy	Rice Hispa Damage	Leaf Blast
Xception	0.856 ± 0.110	0.954 ± 0.026	0.824 ± 0.110	0.702 ± 0.294
ResNet50	0.764 ± 0.173	0.632 ± 0.314	0.533 ± 0.232	0.359 ± 0.278
MobileNet	0.825 ± 0.197	0.937 ± 0.048	0.931 ± 0.062	0.704 ± 0.243
VGG16	0.712 ± 0.414	0.629 ± 0.488	0.600 ± 0.548	0.563 ± 0.519
InceptionV3	0.647 ± 0.185	0.887 ± 0.097	0.563 ± 0.272	0.540 ± 0.306
DenseNet121	0.684 ±0.282	0.770 ± 0.275	0.743 ± 0.167	0.784 ± 0.190
VIT	0.980 ±0.009	0.977 ± 0.005	0.961 ± 0.017	0.967 ± 0.021
SANET	**0.995 ± 0.004**	**0.981 ± 0.002**	**0.985 ± 0.0011**	**0.987 ± 0.007**

**Table 4:** Comparison of our method with state-of-the-art methods in terms of complexity.

Model	Parameters (M)	Training Time (min)/Epoch
VGG16	138	6.6
ResNet50	25.6	3.4
DenseNet121	8.062	9.2
MobileNet	13.58	3.5
Inception V3	24.8	5.55
Xception	23.82	7.4
ViT	307	5.7
SANET	28.62	3.8

## Data Availability

The data are contained within the article and/or available from the corresponding author upon reasonable request.
